# Oxidised LDL and Anti-Oxidised LDL Antibodies Are Reduced by Lipoprotein Apheresis in a Randomised Controlled Trial on Patients with Refractory Angina and Elevated Lipoprotein(a)

**DOI:** 10.3390/antiox10010132

**Published:** 2021-01-18

**Authors:** Tina Z. Khan, Adam Hartley, Dorian Haskard, Mikhail Caga-Anan, Dudley J. Pennell, Peter Collins, Mahmoud Barbir, Ramzi Khamis

**Affiliations:** 1 National Heart and Lung Institute, Imperial College London, Guy Scadding Building, Cale Street, London SW3 6LY, UK; T.Khan@rbht.nhs.uk (T.Z.K.); DJ.Pennell@rbht.nhs.uk (D.J.P.); Peter.collins@imperial.ac.uk (P.C.); 2 Royal Brompton and & Harefield NHS Foundation Trust, Sydney Street, London SW3 6NP, UK; m.barbir@rbht.nhs.uk; 3 National Heart and Lung Institute, Hammersmith Campus, Imperial College London, Du Cane Road, London W12 0NN, UK; adam.hartley12@imperial.ac.uk (A.H.); d.haskard@imperial.ac.uk (D.H.); m.caga-anan@imperial.ac.uk (M.C.-A.); 4 Cardiology Department, Harefield Hospital, Hill End Road, Harefield UB9 6JH, UK

**Keywords:** lipoprotein(a), oxidised LDL, lipoprotein apheresis

## Abstract

**Aims:** An abundance of epidemiological evidence demonstrates that elevated lipoprotein(a) (Lp(a)) represents a significant contributing risk factor towards the development of cardiovascular disease. In particular, raised Lp(a) may play a mechanistic role in patients with refractory angina. Studies have also shown a correlation between oxidised LDL (oxLDL) levels and atherosclerotic burden as well as rates of cardiovascular events. Antibodies against oxLDL (anti-oxLDL) are involved in the removal of oxLDL. Lipoprotein apheresis (LA), which removes lipoproteins using extra-corporeal processes, is an established means of reducing Lp(a), and thereby reduces cardiovascular events. The aim of this study was to investigate the effect of LA on oxLDL and anti-oxLDL levels amongst those with refractory angina in the context of raised Lp(a). **Methods:** We performed a sub-study within a randomised controlled crossover trial involving 20 patients with refractory angina and raised Lp(a) > 500 mg/L, comparing the effect of three months of blinded weekly LA or sham, followed by crossover to the opposite study arm. We utilized enzyme-linked immunosorbent assays (ELISA) to quantify oxLDL and IgG/ IgM anti-oxLDL antibody levels at baseline and following three months of active LA or sham sessions. **Results:** Following three months of LA, there was a 30% reduction in oxLDL from 0.37 ± 0.06 to 0.26 ± 0.04 with a mean drop of −0.11 units (U) (95% CI −0.13, −0.09) compared to no significant change with sham therapy (*p* < 0.0001 between treatment arms). LA also led to a 22% reduction in levels of IgG and IgM anti-oxLDL, again with no significant change demonstrated during sham (*p* = 0.0036 and *p* = 0.012, respectively, between treatment arms). **Conclusion:** Amongst patients with refractory angina in the context of elevated Lp(a), LA significantly lowers levels of oxLDL and anti-oxLDL antibodies, representing potential mechanisms by which LA yields symptomatic and prognostic benefits in this patient cohort.

## 1. Introduction

Based on abundant high quality epidemiological evidence, it is well established that lipoprotein(a) (Lp(a)) is an independent cardiovascular risk factor and predictor of major adverse cardiovascular events [1]. Lipoprotein apheresis (LA) is currently the most effective approved treatment available for raised Lp(a), with minimal effects conferred by conventional lipid lowering agents [2].

Lp(a) has been shown to bind pro-inflammatory-oxidized phospholipids and is a preferential carrier of oxidized phospholipids in human plasma, which may represent one of the numerous mechanisms by which raised Lp(a) may lead to cardiovascular risk [3].

Studies measuring circulating oxidised LDL (oxLDL) have demonstrated its association with atherosclerotic disease [4] and a positive correlation between raised levels of oxLDL and the severity of acute coronary syndromes has been demonstrated [5].

Very few studies have explored the impact of LA on oxLDL and antibodies against oxLDL (anti-oxLDL), none of which were randomised controlled studies. One study assessed the impact of LA in a single individual with severe combined hyperlipidaemia who underwent LA biweekly [6]. Autoantibodies against oxLDL were reduced during the treatment, indicating a lower level of the atherogenic antigen. The authors proposed that this represented an additional benefit of LA therapy [6]. Another study investigated LDL oxidation in eight homozygous patients with familial hypercholesterolemia undergoing LA, and demonstrated that LDL lipid peroxidation, lipid peroxides, and MDA showed an increased resistance against oxidation after repeated LA [7]. The authors concluded that LA not only decreases LDL levels, but also causes changes that render LDL less susceptible to oxidation, and proposed that these changes may contribute to reducing coronary atherosclerosis [7].

More recently, researchers assessed acute changes of systemic oxidative stress biomarkers in patients undergoing apheresis using four different techniques: heparin-induced extracorporeal LDL precipitation (HELP), direct adsorption of lipoproteins (DALI), lipidfiltration (LF), and immunoadsorption of lipoproteins (IA) [8]. All LA methods were similarly effective in lowering LDL cholesterol, oxLDL, and levels of anti-oxLDL antibodies [8].

A further study examined the chronic effects of LA on clinical parameters and endothelial cell function in 19 haemodialysis patients who had peripheral artery disease. In those with an improved ankle brachial pressure index (ABPI) (*n* = 10) [9], apheresis resulted in a long-term reduction of circulating levels of oxLDL, C-reactive protein, and fibrinogen [9]. The authors concluded that LA decreases oxLDL and inflammation and improves endothelial cell function, proposing that this may be one of the mechanisms involved in the long-term therapeutic effect of LA [9].

These observational non-randomised studies suggest that LA-related changes in oxLDL may have a role to play in the therapeutic benefit of LA beyond LDL reduction. None of these studies performed to date specifically assessed the impact of LA on levels of oxLDL and anti-oxLDL antibodies in patients with refractory angina and raised Lp(a). This is a clinically relevant question because Lp(a) has also been shown to bind pro-inflammatory-oxidized phospholipids and is a preferential carrier of oxidized phospholipids in human plasma, which may represent one of the mechanisms by which raised Lp(a) may increase cardiovascular risk. We therefore felt that patients with refractory angina due to progressive cardiovascular disease in the context of elevated Lp(a) represented a clinically relevant group of patients to study. In addition, we wanted to fill a gap in the evidence base by performing a prospective randomised controlled study with a sham placebo control arm to increase the validity of our findings.

We therefore performed a prospective, single-blind, sham-controlled crossover trial in 20 patients with refractory angina and elevated Lp(a) ≥ 50 mg/dL, randomised to three months of weekly lipoprotein apheresis or sham procedures. Our study demonstrated that lipoprotein apheresis resulted in significant improvements in quantitative myocardial perfusion, atheroma burden, exercise capacity, and symptoms including angina, with no significant change observed in the sham treatment arm [10]. As part of the same study, we also hypothesized that LA would lead to a reduction in levels of oxLDL and anti-oxLDL antibodies.

## 2. Materials and Methods

The National Research Ethics Committee authorised ethical approval and local approval was also obtained from our institution’s research department. Prior to commencing the study, it was officially registered via ClinicalTrials.gov (identifier: NCT01796912). Written informed consent was obtained from all participants in the study. The study protocol conformed to the ethical guidelines of the 1975 Declaration of Helsinki. The trial took place between March 2013 and November 2015 at Royal Brompton and Harefield NHS Foundation Trust.

### 2.1. Study Design

This was a randomised, single-blind, sham-controlled, crossover trial performed on 20 patients who had refractory angina in the context of elevated Lp(a) > 50 mg/dL to evaluate the impact of lipoprotein apheresis versus sham on myocardial perfusion reserve and numerous clinical outcomes. Here we present data examining the effect on oxLDL and anti-oxLDL antibodies.

**Figure 1:**
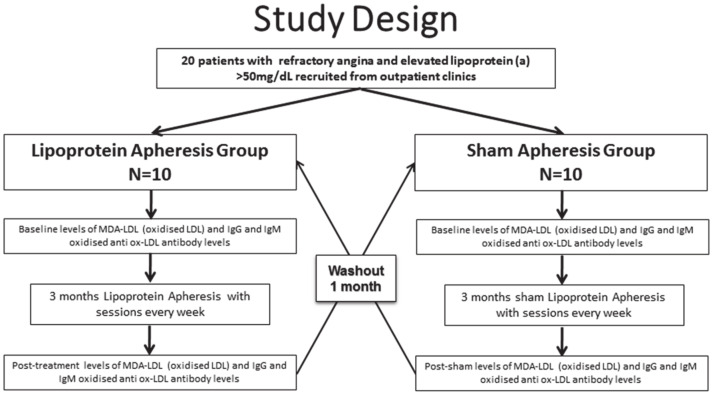
Flow chart summarising the trial methodology.

Participants were randomised to either an initial treatment arm that consisted of weekly lipoprotein apheresis treatment sessions for three months, or to an initial control arm that consisted of weekly sham apheresis sessions for three months (Figure 1). In between crossover to the opposite treatment arm, there was a one-month washout period during which no investigations were performed. The same baseline and post-intervention investigations were repeated prior to and following each three-month treatment period to allow net comparison [11]. Our primary endpoint of quantitative myocardial perfusion assessed with stress perfusion cardiovascular magnetic resonance imaging, as well as carotid atheroma burden, significantly improved with lipoprotein apheresis, with no significant change observed during sham [10]. Secondary endpoints of angina as assessed with the Seattle Angina questionnaire, quality-of-life physical component summary assessed by the short-form 36 survey, and exercise capacity assessed with the six-minute walk test also significantly improved with lipoprotein apheresis, with no significant changes occurring during sham [10].

### 2.2. Patient Population

Patients with refractory angina (defined as angina resistant to optimal medical therapy and unamenable to further revascularisation) were identified and recruited from outpatient cardiology clinics. Refractory angina was confirmed by two cardiologists, who concurred on the absence of revascularisation options and agreed that the pain was ischaemic in nature [11]. The inclusion criterion was refractory angina for a minimum of three months causing a minimum of 2 weekly anginal episodes. Evidence of significant coronary disease was required, fulfilling one or more of these criteria: previous percutaneous coronary revascularisation, coronary bypass surgery, or myocardial infarction. Patients needed to have optimisation of their medical angina therapy that included at least two anti-anginal drugs. All participants had hypercholesterolaemia with elevated Lp(a) > 50 mg/dL and LDL-cholesterol < 4.0 mmol/L, in spite of maximally tolerated lipid-lowering therapy. Patients were excluded if they had suboptimal peripheral venous access, alternative chronic systemic conditions such as liver or renal failure, cancer, significant cardiac failure, severe unstable resting chest pain, recent coronary revascularisation or acute coronary syndrome occurring within eight weeks preceding commencement of the trial, pregnancy, untreated diabetes mellitus, untreated hypertension, and those with contraindications to adenosine or cardiac MRI (CMR) [11].

### 2.3. Randomisation and Blinding

Computer-generated block randomisation was performed using Stata Statistical Software (Version 14.2 by StataCorp, Texas, USA) and patients were randomised 1:1 to start with either the active arm or the sham arm. Throughout the entire protocol all trial participants were kept strictly blinded to the treatment allocation with no breaches [11].

### 2.4. Lipoprotein Apheresis

The DX21 (Direct Hemo Perfusion) DHP Lipoprotein Apheresis machine utilising the Liposorber DL-75 column was used to perform the LA treatments. This LA system binds Apolipoprotein(B) (ApoB)-containing lipoproteins directly from whole blood using dextran sulphate in the treatment column [11]. Sham apheresis sessions were performed in the control group with needle insertion; however, tubing was not connected to the machine and the apheresis machine was run to simulate active treatment using dummy columns. Patients were blinded to the treatment allocation with the use of customised screens and drapes [11].

### 2.5. Assessment of Lipid Parameters

At the four data collection time points in the trial (pre- and post-apheresis and pre-and post-sham), blood tests were drawn for the full lipid profile, including total cholesterol (TC), Lp(a) using the Ultra latex immunoassay, LDL cholesterol, HDL cholesterol, total cholesterol-to-HDL ratio (TC:HDL), triglycerides (TG); apolipoprotein(A) (Apo(A)) and Apo(B), and oxLDL and anti-oxLDL antibodies. Pre-apheresis and pre-sham blood tests were drawn just prior to commencing the first apheresis or sham session. Post-apheresis and post-sham blood tests were drawn at least one hour after completing the final apheresis or sham treatment session via fresh venepuncture to avoid haemodilution errors.

### 2.6. Generation of MDA-LDL

MDA-LDL was prepared as described previously [12]. Briefly, native LDL was incubated with 0.5 M MDA solution at 37 °C for 3 h, prepared through acid hydrolysis of MDA-bis-dimethylacetal (Sigma-Aldrich, Poole, UK). Following modification, the MDA-LDL conjugate was then eluted with phosphate-buffered saline (PBS) through a PD-10 column and 0.01% EDTA was added to prevent further oxidation.

### 2.7. Enzyme-Linked Immunosorbent Assay (ELISA) to Detect MDA-LDL

All samples were anonymised and personnel conducting assays were fully blinded to the patient treatment allocation. ELISAs to detect MDA-LDL and anti-oxLDL antibodies were performed as previously described [13]. Levels of MDA-LDL were measured using 10 μg/mL LO1, a novel laboratory-developed monoclonal IgG3κ murine antibody, as the capture antibody in a sandwich enzyme-linked immunosorbent assay (ELISA). LO1 reacts with MDA-LDL but minimally with native LDL [14]. A biotinylated anti-ApoB antibody (Abcam, Cambridge, MA, USA) at 1:2000 dilution and horseradish peroxidase (HRP)-conjugated streptavidin (R&D Systems, Minneapolis, MN, USA) at 1:200 dilution were used for detection. Subsequently, for this and all other ELISAs, 3,3′,5,5′-tetramethylbenzidine (TMB) (Sigma Aldrich, Poole, UK) was added and the reaction stopped with 0.5 M H2SO4. Plates were read at an optical density (OD) of 450 nm using a Synergy HT microplate reader (BioTek, Winooski, VT, USA).

### 2.8. ELISA to Detect Anti-oxLDL and Total IgG/IgM Antibodies

IgG and IgM antibody binding to solid phase antigens was identified by mouse anti-human IgG (Cambridge Bioscience, Cambridge, UK) or biotinylated mouse anti-human IgM (Cambridge Bioscience) followed by HRP-conjugated rabbit anti-mouse Ig (Dako, Cambridgeshire, UK) or HRP-conjugated streptavidin (R&D Systems, Minneapolis, MN, USA) in indirect ELISA format.

Total IgG and IgM antibody levels were measured using capture ELISA. Goat anti-human IgG or mouse anti-human IgM (Southern Biotech, Birmingham, AL, USA) were used to capture IgG or IgM, respectively. Detection antibodies were biotinylated goat F(ab′)_²_ anti-human IgG or biotinylated mouse anti-human IgM (both Southern Biotech).

### 2.9. Statistical Analysis

GraphPad Prism 6 (La Jolla, San Diego, CA, USA) was used for statistical analysis. For MDA-LDL and total IgG/IgM, log transformed standard curve optical density values were fitted, which were then used to interpolate sample concentrations from sample OD values. Raw OD values were analysed for anti-oxLDL antibodies. Paired t-tests were used for normally distributed data and the Wilcoxon signed-rank test was used for non-normally distributed data. Statistical significance was defined as a *p*-value < 0.05.

## 3. Results

**Figure 2:**
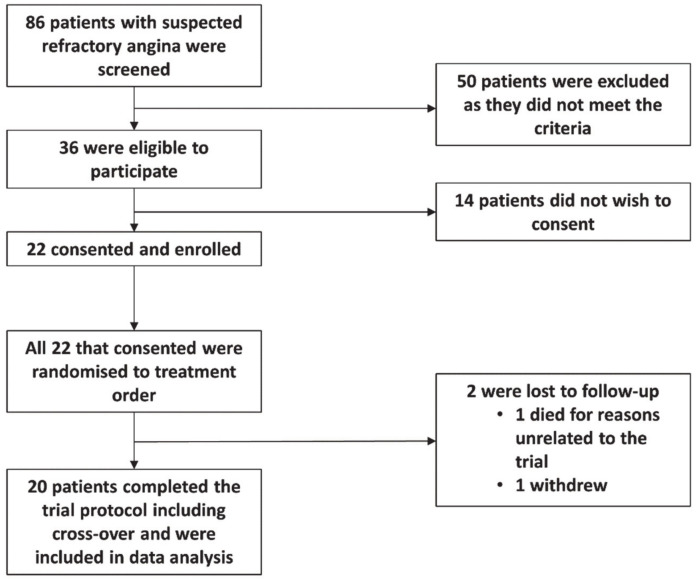
CONSORT flow chart.

In total 20 patients completed the trial protocol (Figure 2). Table 1 depicts the baseline characteristics of the participants according to the order in which treatment was initially randomised. One patient needed to be switched to a double filtration HF440 lipoprotein apheresis system given that they were unable to tolerate the DX 21 DHP system.

### 3.1. Changes in Oxidised LDL and Their Antibodies

MDA-LDL (OD405 nm) reduced by a mean value of −0.11 (95% CI −0.13, −0.09) during apheresis from 0.37 ± 0.06 to 0.26 ± 0.04. During sham, it did not change significantly, with a mean change of −0.01 (95% CI −0.04, 0.02) from 0.35 ± 0.07 to 0.34 ± 0.07 (*p* < 0.0001 between groups) (Table 2 and Table 3).

IgG anti-MDA-LDL (OD450 nm) reduced by a median value of −0.14 (IQR −0.19, −0.06) during apheresis from 0.61 ± 0.21 to 0.47 ± 0.20. During sham, it did not change significantly, with a median change of −0.008 (IQR −0.060, 0.03) from 0.57 ± 0.21 to 0.55 ± 0.21 (*p* = 0.0036 between groups) (Table 2 and Table 3).

Total IgG (g/L) reduced by a median value of −2.45 (IQR −3.71, −0.94) during apheresis from 13.54 ± 3.89 to 11.13 ± 3.84. During sham, it did not change significantly, with a median change of −0.05 (IQR −1.05, 0.57) from 13.68 ± 5.40 to 13.35 ± 3.96 (*p* = 0.0019 between groups) (See Table 2 and Table 3).

IgM anti-MDA-LDL (OD450 nm) reduced by a median value of −0.15 (IQR −0.26, −0.04) during apheresis from 0.66 ± 0.43 to 0.54 ± 0.36. During sham, it did not change significantly, with a median change of −0.015 (IQR −0.08, 0.06) from 0.67 ± 0.39 to 0.67 ± 0.44 (*p* = 0.012 between groups) (Table 2 and Table 3).

Total IgM (g/L) reduced by a median value of −0.20 (IQR −0.32, −0.08) during apheresis from 0.77 (IQR 0.43, 0.98) to 0.61 (IQR 0.36, 0.68). During sham, it did not change significantly, with a median change of −0.02 (IQR −0.03, 0.01) from 0.72 (IQR 0.47, 0.93) to 0.71 (IQR 0.48, 1.03) (*p* = 0.0009 between groups) (Table 2 and Table 3).

### 3.2. Changes in Lp(a) and Conventional Lipid Parameters

Lp(a) (mg/L) reduced by a median value of −679.5 (IQR −1102, −453) during apheresis from 1001.5 (IQR 695.5, 1429.5) to 248.0 (IQR 171.5, 339.5). During sham, it did not change significantly, with a median change of −5.5 (IQR −48.85, 51.5) from 942.5 (IQR 686.5, 1441.0) to 885.5 (IQR 633.5, 1467.0) (*p* = 0.0001 between groups) (Table 2 and Table 3).

TC (mmol/L) reduced by a median value of −1.7 (IQR −2.2, −1.5) during apheresis from 3.67 ± 0.81 to 1.84 ± 0.39. During sham, it did not change, with a median change of 0.1 (IQR −0.25, 0.30) from 3.78 ± 1.05 to 3.74 ± 0.73 (*p* = 0.0001 between groups) (Table 2 and Table 3).

LDL cholesterol (mmol/L) reduced by a median value of −1.55 (IQR −1.90, −1.17) during apheresis from 1.99 ± 0.68 to 0.40 ± 0.27. During sham, it did not change, with a median change of −0.025 (IQR −0.035, 0.065) from 2.14 ± 0.91 to 2.01 ± 0.63 (*p* = 0.0001 between groups) (Table 2 and Table 3).

HDL cholesterol (mmol/L) reduced slightly by a mean value of −0.124 (95% CI −0.213, −0.035) during apheresis from 1.11 ± 0.28 to 0.99 ± 0.23. During sham, it did not change, with a mean change of −0.0015 (95% CI −0.077, 0.073) from 1.14 ± 0.29 to 1.14 ± 0.27 (*p* = 0.006 between groups) (Table 2 and Table 3).

TC:HDL reduced by a median value of −1.635 (IQR −1.75, −1.235) during apheresis from 3.42 ± 0.87 to 1.89 ± 0.39. During sham, it did not change, with a median change of 0.025 (IQR −0.275, 0.350) from 3.46 ± 0.94 to 3.41 ± 0.77 (*p* = 0.0002 between groups) (Table 2 and Table 3).

TG (mmol/L) reduced moderately by a mean value of −0.28 (95% CI −0.49, −0.07) during apheresis from 1.22 ± 0.48 to 0.94 ± 0.45. During sham, there was no significant change, with a mean change of 0.18 (95% CI −0.02, 0.37) from 1.18 ± 0.38 to 1.36 ± 0.48 (*p* = 0.007 between groups) (Table 2 and Table 3).

ApoA (g/L) reduced very slightly by a mean value of −0.085 (95% CI −0.166, −0.004) during apheresis from 1.24 ± 0.24 to 1.15 ± 0.19. During sham, it did not change, with a mean change of −0.01 (95% CI −0.055, 0.035) from 1.27 ± 0.19 to 1.26 ± 0.20 (*p* = 0.074 between groups) (Table 2 and Table 3).

ApoB (g/L) reduced by a mean value of −0.41 (95% CI −0.47, −0.34) during apheresis from 0.80 (IQR 0.65, 0.90) to 0.4 (IQR 0.4, 0.4). During sham, it did not change, with a mean change of −0.04 (95% CI −0.11, 0.03) from 0.77 (IQR 0.70, 0.89) to 0.75 (IQR 0.68, 0.87) (*p* = 0.0001 between groups) (Table 2 and Table 3).

## 4. Discussion

This was the first prospective randomised controlled study to assess the effects of lipoprotein apheresis on levels of oxLDL and anti-oxLDL antibodies in patients with elevated Lp(a) and refractory angina.

MDA-LDL reduced by a mean of 30% of the baseline value after three months of apheresis, with no change during sham and with statistical significance (*p* < 0.0001 between groups). This could be due to a combination of reduced levels of its most potent carrier Lp(a) with apheresis, and because it may be directly removed itself via adsorption to the apheresis column. Moreover, all measured antibodies reduced during three months of apheresis and did not change during sham. This could be due to lower levels of their associated antigen (MDA-LDL) or because the complexes they form with MDA-LDL may be directly removed via adsorption to the apheresis column.

It was shown that a significant amount of oxLDL accumulates in atherosclerotic plaques [15,16]. As described previously, Matsuo et al. found that MDA-LDL levels were associated with thin cap fibroatheromas as detected by optimal coherence tomography, suggesting that MDA-LDL could be regarded as a marker of plaque vulnerability [4]. Therefore, albeit speculative, it is possible that reduction in MDA-LDL could represent yet another mechanism by which there may be reduction in the risk of spontaneous myocardial infarctions arising from unstable plaques. Furthermore, a positive relationship between raised levels of oxLDL and the severity of acute coronary syndromes was demonstrated [5], implicating a potential benefit of apheresis-induced reduction in oxidised LDL. In the future, prospective randomised controlled studies assessing the impact of oxLDL reduction on cardiovascular events can help to establish the potential therapeutic role of lowering oxLDL in the sequelae of cardiovascular disease.

The role of IgG and IgM anti-oxLDL antibodies is less clear. On the one hand, there is a correlation between the existence and titres of anti-oxLDL antibodies and the extent of atherosclerosis and cardiovascular disease [17]. Conversely, experimental data indicate that anti-oxLDL antibodies may potentially be protective [17]. Although it is difficult to conclude whether dynamic changes in IgG anti-MDA-LDL are beneficial or pathogenic, there is now mounting evidence that higher levels of IgM anti-MDA-LDL may confer protection from adverse atherosclerotic plaque components, such as a necrotic core, and indeed cardiovascular events [13]. Thus, the reduction in antibody titres seen with LA may not itself be a favourable feature.

There is increasing evidence that inflammatory processes play an important role in cardiovascular disease, with the recent Canakinumab Anti-Inflammatory Thrombosis Outcome Study (CANTOS) Trial showing that anti-inflammatory therapy with canakinumab, targeting the interleukin-1β innate immunity pathway, led to a significantly lower rate of recurrent cardiovascular events compared to placebo, which was independent of the degree of lipid-level lowering [18]. Regarding the present study, uncertainty remains regarding the mechanism of oxLDL reduction, which may either be simply mechanically removed by the apheresis column, or apheresis may also reduce inflammatory load via an alternative mechanism, thereby reducing the formation of oxidised LDL. Although it is not within the scope of the present study, in retrospect measurement of inflammatory markers such as high sensitivity C reactive protein (hs-CRP), interleukins IL-1β/IL-6, or myeloperoxidase would have helped to address whether the inflammatory load was also modified by this treatment.

Lp(a) reduced by a median value of 68% of the baseline value after three months of apheresis, with no significant change during sham (*p* = 0.0001 between groups). The reduction achieved in the Lp(a) Ultra latex immunoassay is consistent with previously documented reductions of Lp(a) via apheresis using dextran sulphate columns, in which Lp(a) reduced by 67.4% +/−11.6% immediately following apheresis [19]. A recent review article stated that apheresis can acutely decrease Lp(a) by approximately 60–75%, which is also consistent with our data [20].

LDL cholesterol reduced by a median value of 78% of the baseline value after three months of apheresis, with no change during sham (*p* = 0.0001 between groups). This degree of reduction in fact superseded the acute reductions in LDL cholesterol achieved with dextran sulphate-based systems reported previously in the literature [19,21]. There are two potential explanations for this discrepancy. Firstly, the acute reductions in LDL cholesterol reported in the literature used the same systems reported after a single treatment only, unlike the three month therapy used here [19,21]. Secondly, the baseline pre-treatment LDL cholesterol in our study was intentionally relatively low, at 2.16 ± 0.73 mmol/L. In comparison, the mean pre-treatment LDL cholesterol reported in the literature was significantly higher, at 4.81 ± 0.72 mmol/L [21], as the majority of patients assessed had familial hypercholesterolaemia, making comparisons problematic.

### Study Limitations

Our study is obviously limited by its small sample size. The heterogenous nature of the patient characteristics represents another limitation; however, the crossover design of the study ensured that patients effectively acted as self-controls, allowing valid comparisons [11]. Another question that remains unresolved is how long the effects of LA on oxLDL and their associated antibodies last, and whether there is sustained benefit or whether a rebound phenomenon occurs. The study compared samples taken before the first apheresis or sham procedure versus samples taken after three months following the final apheresis or sham procedure, without interim sampling of oxLDL and their associated antibodies before and after individual treatments. Therefore, the study design does not allow one to distinguish between long-term changes in lipid parameters versus changes caused by a single procedure. In addition, analysis of the content of the post-treatment apheresis columns, which was not performed within this study, may have allowed us to quantify the direct removal of oxLDL levels and anti-oxLDL antibodies. The relevance of the raised baseline Lp(a) levels in the study participants with respect to the degree of LA-related reduction in oxLDL levels and anti-oxLDL antibodies also remains uncertain. In future, it may be interesting to repeat this study in patients with refractory angina with normal Lp(a) levels to assess whether a similar reduction in oxLDL levels, anti-oxLDL antibodies, and clinical endpoints is achieved, which, if present, would imply that these observed LA-related benefits are independent of a raised baseline Lp(a). Finally, we cannot prove causation and conclude that reduction in oxLDL improves cardiovascular outcomes because it is beyond the scope of this study and merits further investigation.

## 5. Conclusions

In patients with refractory angina in the context of elevated Lp(a), LA significantly lowers levels of oxLDL levels and anti-oxLDL antibodies, representing potentially important mechanisms by which LA yields symptomatic and prognostic benefits in this patient cohort. Prospective randomised controlled studies assessing the impact of oxLDL reduction on cardiovascular events are required to establish the potential therapeutic role of lowering oxLDL in cardiovascular disease.

## Figures and Tables

**Table 1:** Demographic and clinical characteristics at baseline.

Variable	Apheresis/Sham	Sham/Apheresis	All Subjects
n	9	11	20
Age in Years	59.1 (10.4)	62.4 (9.0)	60.9 (9.5)
Male	9 (100)	10 (91)	19 (95)
Ethnicity: White Asian	4 (44.4) 5 (55.6)	3 (27.3) 8 (72.7)	7 (35.0) 13 (65.0)
Body Mass Index (kg/m^2^)	27.3 (1.9)	27.5 (4.1)	27.4 (3.2)
Diabetes	2 (22.2)	1 (9.1)	3 (15.0)
Hypertension	4 (44.4)	8 (72.7)	12 (60.0)
Smoking: Never Ex Current	3 (37.3) 4 (44.4) 2 (22.2)	7 (63.6) 2 (18.2) 2 (18.2)	10 (50.0) 6 (30.0) 4 (20.0)
Family History of CAD	7 (77.8)	9 (81.8)	16 (80.0)
Prior Coronary Artery Bypass Graft Surgery	6 (66.7)	6 (54.6)	12 (60.0)
Prior percutaneous Coronary Intervention	7 (77.8)	9 (81.8)	16 (80.0)
Prior Myocardial Infarction	8 (88.9)	9 (81.8)	17 (85.0)
Anti-anginal Drugs			
Oral Nitrate	7 (77.8)	7 (63.6)	14 (70.0)
Beta blocker	7 (77.8)	11 (100)	18 (90.0)
Calcium Channel Blocker	3 (33.3)	5 (45.5)	8 (40.0)
Ivabradine	2 (22.2)	2 (18.2)	4 (20.0)
Ranolazine	1 (11.1)	0	1 (5.0)
Statin	9 (100.0)	11 (100.0)	20 (100.0)
Anti-platelet Agents	9 (100.0)	11 (100.0)	20 (100.0)
Oral Anticoagulants	1 (11.1)	3 (27.3)	4 (20)
Systolic Blood Pressure (mmHg)	125.6 (8.5)	125.5 (9.1)	125.5 (8.6)
Diastolic Blood Pressure (mmHg)	72.2 (9.4)	71.4 (2.3)	71.8 (6.3)
Baseline Lp(a) (mg/dL)	112.0 (77.1, 166.0)	108.0 (90.2, 152)	110.0 (77.1, 159)
Total Cholesterol (mmol/L)	3.46 (0.82)	4.25 (0.74)	3.90 (0.86)
LDL Cholesterol (mmol/L)	1.85 (0.74)	2.41 (0.64)	2.16 (0.73)
HDL Cholesterol (mmol/L)	1.11 (0.31)	1.11 (0.28)	1.11 (0.28)
TG Cholesterol (mmol/L)	1.22 (0.54)	1.22 (0.45)	1.22 (0.48)
Haemoglobin (g/L)	135.1 (16.9)	134.9 (12.4)	135.0 (14.2)
Platelet Count x 10^9^/L	294.11 (36.92)	194.45 (28.11)	198.80 (31.85)

Data are mean (SD), *n* (%), median (interquartile range). LDL = low-density lipoprotein, HDL = high-density lipoprotein, TG = triglyceride, CAD = coronary artery disease.

**Table 2:** Change in MDA-LDL and its associated antibodies and lipid parameters during apheresis and sham shown as mean (lower 95% CI, upper 95% CI) or median IQR.

Variable	Apheresis	Sham	*p* (Between Groups)
MDA-LDL Citrate (OD405 nm)	−0.11 (−0.13, −0.09)	−0.01 (−0.04, 0.02)	<0.0001
IgG anti-MDA-LDL (OD450 nm)	−0.14 [−0.19, −0.06]	−0.008 [−0.060, 0.03]	0.0036
Total IgG (g/L)	−2.45 [−3.71, −0.94]	−0.05 [1.05, 0.57]	0.0019
IgM anti-MDA-LDL (OD450 nm)	−0.15 [−0.25, −0.04]	−0.015 [−0.076, 0.055]	0.012
Total IgM (g/L)	−0.20 [−0.32, −0.08]	0.02 [−0.03, 0.01]	0.0009
Lp(a) Image Assay (mg/L)	−903 [−1474, −513]	−66 [−138, 170]	0.0001
Lp(a) Ultra Latex Assay (mg/L)	−679.5 [−1102, −453]	−5.5 [−48.85, 51.5]	0.0001
Total Cholesterol (mmol/L)	−1.7 [−2.2, −1.5]	0.1 [−0.25, 0.30]	0.0001
LDL Cholesterol (mmol/L)	−1.55 [−1.90, −1.17]	−0.03[−0.04, 0.07]	0.0001
HDL Cholesterol (mmol/L)	−0.12 (−0.21, −0.04)	−0.002 (−0.08, 0.07)	0.006
TC:HDL ratio	−1.635 [−1.75, −1.24]	0.025 [−0.28, 0.35]	0.0002
Triglycerides (mmol/L)	−0.28 (−0.49, −0.07)	0.18 (−0;02, 0.37)	0.007
Apolipoprotein A (g/L)	−0.09 (−0.17, −0.00)	−0.01 (−0.06, 0.04)	0.074
Apolipoprotein B (g/L)	−0.41 (−0.47, −0.34)	−0.04 (−0.11, 0.03)	<0.0001

Lp(a) = Lipoprotein(a), LDL = low density lipoprotein, HDL = high density lipoprotein.

**Table 3:** Absolute levels of MDA-LDL and their associated antibodies and lipid parameters before and after apheresis and sham shown as mean ± SD or median [IQR].

Variable	Pre-Apheresis	Post-Apheresis	Pre-Sham	Post-Sham
MDA-LDL Citrate (OD405 nm)	0.37 ± 0.06	0.26 ± 0.04	0.35 ± 0.07	0.34 ± 0.07
IgG anti-MDA-LDL (OD450 nm)	0.61 ± 0.21	0.47 ± 0.20	0.57 ± 0.21	0.55 ± 0.21
Total IgG (g/L)	13.54 ± 3.89	11.13 ± 3.84	13.68 ± 5.40	13.35 ± 3.96
IgM anti-MDA-LDL (OD450 nm)	0.66 ± 0.43	0.54 ± 0.36	0.67 ± 0.39	0.67 ± 0.44
Total IgM (g/L)	0.77 [0.43, 0.98]	0.61 [0.36, 0.68]	0.72 [0.47, 0.93]	0.71 [0.48,1.03]
Lp(a) Ultra Latex Assay (mg/L)	1001.5 [695.5, 1429.5]	248.0 [171.5, 339.5]	942.5 [686.5, 1441.0]	885.5 [633.5, 1467.0]
Total Cholesterol (mmol/L)	3.67 ± 0.81	1.84 ± 0.39	3.78 ± 1.05	3.74 ± 0.73
LDL Cholesterol (mmol/L)	1.99 ± 0.68	0.40 ± 0.27	2.14 ± 0.91	2.01 ± 0.63
HDL Cholesterol (mmol/L)	1.11 ± 0.28	0.99 ± 0.23	1.14 ± 0.29	1.14 ± 0.27
TC:HDL Ratio	3.42 ± 0.87	1.89 ± 0.39	3.46 ± 0.94	3.41 ± 0.77
Triglycerides (mmol/L)	1.22 ± 0.48	0.94 ± 0.45	1.18 ± 0.38	1.36 ± 0.48
Apolipoprotein A (g/L)	1.24 ± 0.24	1.15 ± 0.19	1.27 ± 0.19	1.26 ± 0.20
Apolipoprotein B (g/L)	0.80 [0.65, 0.90]	0.4 [0.4, 0.4]	0.77 [0.70, 0.89]	0.75 [0.68, 0.87]

## Data Availability

Fully anonymised data presented in this study can potentially be available upon specific request at the discretion of the corresponding author. The data are not publicly available for privacy and ethical reasons given that the research participants who consented for the study did not provide specific consent to have their data shared in a public database.
